# Polyphenol-Rich *Euterpe oleracea* Mart. Seed Extract Improves High-Fat-Diet-Induced MASLD Through Modulation of Hepatic Mitochondrial Respiration, Biogenesis, and Redox Homeostasis

**DOI:** 10.3390/molecules31162925

**Published:** 2026-08-21

**Authors:** Dafne L. Beserra-Silva, Julia F. Gouveia, Beatriz C. de Oliveira, Mariana A. Cavalheira, Natália P. A. Nogueira, Marcia Cristina Paes, Simone V. da Silva, Ana Lucia R. Nascimento, Jorge José de Carvalho, Dayane T. Ognibene, Cristiane A. da Costa, Graziele F. de Bem, Angela C. Resende

**Affiliations:** 1 Department of Pharmacology, Institute of Biology, Rio de Janeiro State University, Rio de Janeiro 20551-030, Brazil; dafnelopes.bs@gmail.com (D.L.B.-S.); juliafg02@gmail.com (J.F.G.); beatrizcardoso849@gmail.com (B.C.d.O.); alencar.mariana@uerj.br (M.A.C.); dayane.ognibene@uerj.br (D.T.O.); cristiane.aguiar.costa@uerj.br (C.A.d.C.); bem.graziele@uerj.br (G.F.d.B.); 2 Department of Biochemistry, Institute of Biology, Rio de Janeiro State University, Rio de Janeiro 20551-030, Brazil; natalia.nogueira@uerj.br (N.P.A.N.); mcpaes@uerj.br (M.C.P.); 3 Department of Cell Biology, Institute of Biology, Rio de Janeiro State University, Rio de Janeiro 20551-030, Brazil; simone.vargas.silva@uerj.br; 4 Department of Histology and Embryology, Institute of Biology, Rio de Janeiro State University, Rio de Janeiro 20551-030, Brazil; ana.nascimento@uerj.br (A.L.R.N.); carvalho@uerj.br (J.J.d.C.)

**Keywords:** *Euterpe oleracea* Mart., exercise, liver, mitochondria, oxidative stress

## Abstract

Metabolic dysfunction-associated steatotic liver disease (MASLD) is the most prevalent chronic liver disease, characterized by hepatic steatosis, mitochondrial dysfunction, and oxidative stress. Food-derived polyphenols are promising ingredients that modulate cellular metabolism and redox balance. Açaí (*Euterpe oleracea* Mart.) seed, a polyphenol-rich agro-industrial by-product, has shown hepatoprotective potential; however, no previous study has comprehensively evaluated its effects on key mitochondrial functions in experimental MASLD. Therefore, this study investigated whether açaí seed extract (ASE), administered alone or in combination with physical training, could improve hepatic mitochondrial respiration, biogenesis, ultrastructural integrity, and redox homeostasis in HF diet-fed Sprague–Dawley rats. Male rats were fed a control or HF diet for 16 weeks and treated with ASE (200 mg/kg/day), aerobic training, or both during the final six weeks. ASE reduced hepatic steatosis by approximately 52% compared with the HF group, improved mitochondrial ultrastructure, increased the expression of the mitochondrial biogenesis regulators PGC-1α and NRF-1, with a 3-fold increase in PGC-1α mRNA expression, increased TFAM immunoreactivity, increased PPARα expression, reduced oxidative stress, and restored lipid-driven mitochondrial respiration. Physical training improved glycemic control and mitochondrial structure, with limited effects on mitochondrial function and redox balance. Combined treatment reduced plasma triglycerides by 41%, increased CPT1α expression, and enhanced carbohydrate-supported mitochondrial respiration. These findings provide mechanistic support for further investigation of açaí seed-derived polyphenols as candidates for nutritional strategies targeting MASLD.

## 1. Introduction

Food-derived polyphenols have attracted increasing attention for their ability to modulate metabolic pathways involved in obesity-related disorders, particularly by regulating mitochondrial metabolism, biogenesis, antioxidant defense, and lipid homeostasis, which are key mechanisms underlying metabolic dysfunction-associated steatotic liver disease (MASLD) [1]. Worldwide, MASLD stands as the most common chronic liver disease, with its development being strongly influenced by obesity, excessive nutrient consumption, and low levels of physical activity [2,3,4].

Excessive consumption of a high-fat diet (HFD) promotes hepatic lipid accumulation, mitochondrial dysfunction, oxidative stress, and endoplasmic reticulum stress, impairing fatty acid (FA) oxidation and metabolic homeostasis [5,6,7]. Hepatic lipid utilization and cellular energy production critically depend on mitochondrial oxidative metabolism [1,2]. Therefore, the structural and functional integrity of mitochondria is essential for maintaining cellular bioenergetics and metabolic homeostasis. In this context, mitochondrial dysfunction is a central driver of MASLD and other obesity-associated metabolic disorders [5,8]. Consequently, nutritional strategies that restore mitochondrial function and redox homeostasis have emerged as promising approaches to delay MASLD progression [1].

Açaí is the fruit of the Amazon palm *Euterpe oleracea* Mart. It contains bioactive polyphenols that offer antioxidant and metabolic regulatory properties [9]. Brazil generates about 1.6 million tons of açaí seed waste annually, which places an environmental burden on ecosystems. Most industries have not tapped this waste for circular bioeconomy strategies in agri-food resources [10]. Seeds account for 80–95% of the fruit mass and provide phenolic compounds, offering a sustainable yet underused source of value-added ingredients. Despite this, açaí seeds are mostly discarded as agro-industrial residues during processing [9,11].

Previous phytochemical studies show that açaí seed extract (ASE) is particularly rich in flavan-3-ols, including catechin, epicatechin, and proanthocyanidins [12]. Experimental studies demonstrate that ASE improves obesity-related metabolic alterations in HFD-fed animals and may exert hepatoprotective effects [12]. These findings establish ASE as a promising source of functional food ingredients with potential applications in the prevention of metabolic diseases. Although ASE has shown beneficial effects on obesity-associated metabolic disturbances, it remains unknown whether these properties involve preservation of mitochondrial respiratory function and ultrastructural integrity, regulation of mitochondrial biogenesis, and maintenance of hepatic redox homeostasis during MASLD progression.

Since mitochondrial dysfunction is a central hallmark of MASLD, combining dietary bioactive compounds with non-pharmacological interventions that also target mitochondrial metabolism may provide additive or synergistic metabolic benefits. Physical training is another established strategy for improving metabolic health, enhancing mitochondrial function, and promoting redox balance [13,14,15,16,17]. Both polyphenol-rich extracts and aerobic exercise have been shown to modulate the Peroxisome Proliferator-Activated Receptor γ Coactivator 1-α (PGC-1α) signaling axis, a master regulator of mitochondrial biogenesis and oxidative metabolism [18], providing a strong biological rationale for investigating their combined effects. Moreover, aerobic training is the only non-pharmacological intervention with established clinical efficacy for MASLD management [16], making it a clinically relevant strategy to evaluate alongside a nutraceutical candidate. However, whether aerobic exercise and açaí seed-derived polyphenols exert additive or synergistic effects on hepatic mitochondrial metabolism remains poorly understood.

Therefore, this study investigated the effects of ASE supplementation, alone or combined with moderate physical training, on hepatic mitochondrial metabolism, biogenesis, redox homeostasis, and ultrastructural integrity in rats with HFD-induced MASLD. By elucidating how ASE modulates these processes, this study may provide mechanistic evidence supporting the use of açaí seed-derived polyphenols to prevent or manage MASLD.

## 2. Results

### 2.1. Body Weight Gain, Feed Efficiency, Blood Glucose, and Plasma Lipid Profile

Animals in the HF+T and HF+TA groups showed better physical performance, with no significant differences between them (Figure S1). The HF group showed a marked increase in body mass during the experiment (Table 1) versus the CT group (*p* < 0.0001). All interventions reduced this gain compared to the HF group (HF+A: *p* < 0.0001; HF+T: *p* = 0.0016; HF+TA: *p* < 0.0001), with the combined intervention (HF+TA) more effective than exercise alone (*p* = 0.0051). Feed efficiency (Table 1) was also higher in the HF group than the CT group (*p* < 0.0001). All treatments reduced feed efficiency (HF+A: *p* < 0.0001; HF+T: *p* = 0.0022; HF+TA: *p* < 0.0001), indicating improved metabolic use.

At the end of the intervention, final blood glucose levels were higher in both HF (*p* = 0.0002) and HF+A (*p* = 0.0018) groups compared to CT. Notably, only the HF+T group showed a significant reduction in glycemia relative to both HF (*p* = 0.0041) and HF+A (*p* = 0.0260) groups. Total cholesterol levels were elevated in all HF-fed groups compared to CT (HF: *p* < 0.001; HF+A: *p* = 0.0004; HF+T: *p* = 0.0022; HF+TA: *p* = 0.0012), with no significant differences among them. Triglyceride levels were increased in the HF (*p* = 0.0061) and HF+T (*p* = 0.0128) groups compared to CT. Interestingly, only the combined intervention (HF+TA) significantly reduced triglyceride levels relative to both HF (*p* = 0.0147) and HF+T (*p* = 0.0296), suggesting a synergistic effect.

### 2.2. Hepatic Lipid Content

**Figure 1:**
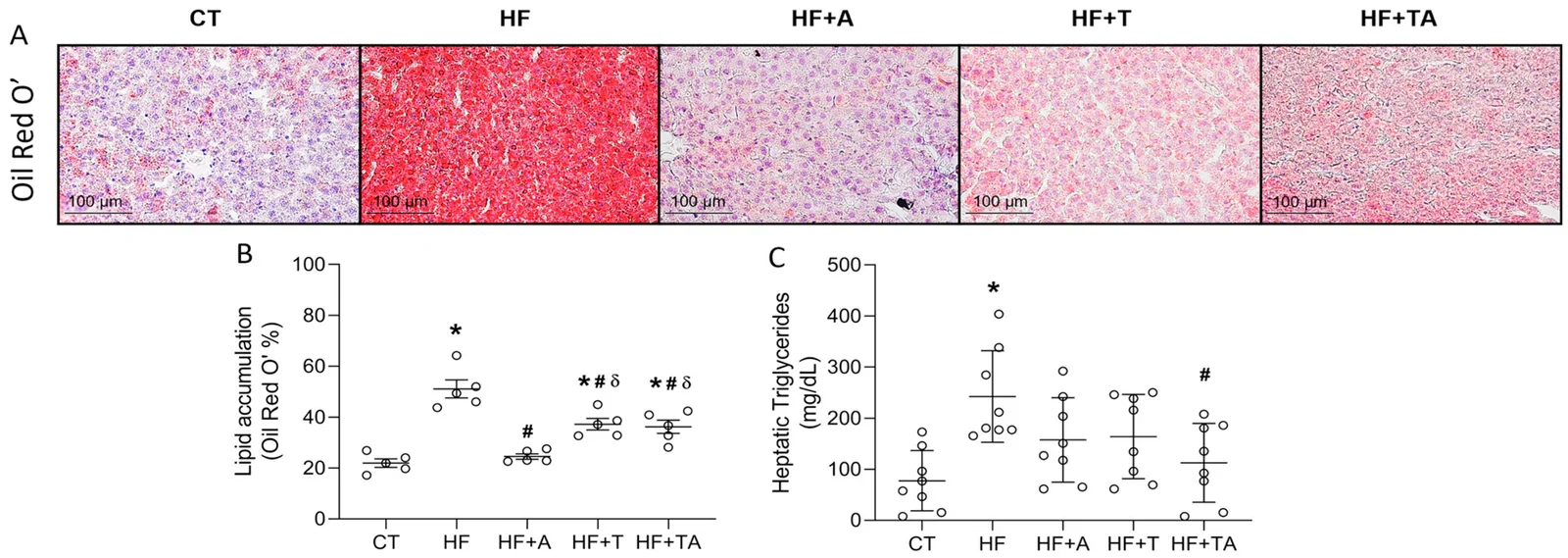
Histological analysis and quantification of hepatic lipid accumulation in high-fat-diet-fed rats following ASE and physical training. (**A**) Representative liver sections stained with Oil Red O (40×; scale bar = 100 µm). (**B**) Hepatic lipid accumulation quantified as the Oil Red O-positive area fraction. (**C**) Hepatic triglyceride levels. Data are presented as mean ± SEM (n = 5 per group for Oil Red O staining; n = 8 per group for hepatic triglycerides). Statistical analysis was performed using one-way ANOVA followed by Tukey’s post hoc test. * *p* ≤ 0.05 vs. CT; # *p* ≤ 0.05 vs. HF; δ *p* ≤ 0.05 vs. HF+A.

The HF group exhibited a marked increase in hepatic lipid accumulation compared to the CT group (*p* < 0.0001), as evidenced by enhanced Oil Red O staining (Figure 1). Treatment with ASE (HF+A) significantly reduced hepatic lipid content compared with the HF group (*p* < 0.0001), restoring levels comparable to those observed in the CT group. Similarly, the HF+T and HF+TA groups showed significant reductions in hepatic lipid accumulation compared to HF (HF+T: *p* = 0.0045; HF+TA: *p* = 0.0022). However, lipid levels in these groups remained significantly higher than those observed in the HF+A group, indicating a comparatively lower efficacy.

Additionally, biochemical analysis confirmed elevated hepatic triglyceride content in the HF group compared to CT (242.4 ± 89.65 mg/dL vs. 77.40 ± 58.92 mg/dL; *p* = 0.0016), with a significant reduction observed only in the combined intervention group (HF+TA: 112.7 ± 77.20 mg/dL; *p* = 0.0182 vs. HF).

### 2.3. Analysis of Liver Ultrastructure and Mitochondria Morphometry

**Figure 2:**
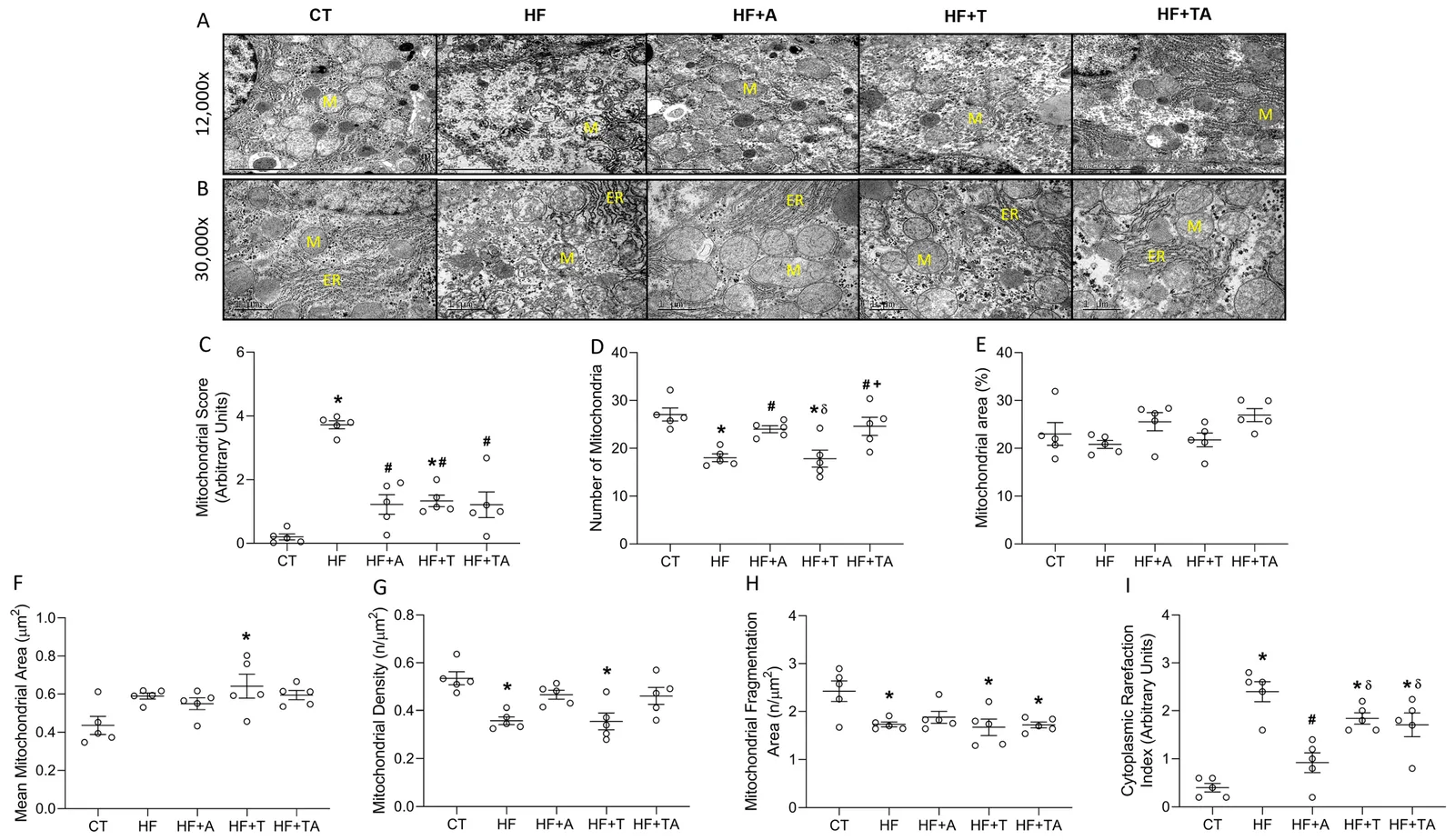
Ultrastructural analysis and quantification of hepatic mitochondrial parameters in high-fat-diet-fed rats following ASE and physical training. Representative transmission electron micrographs acquired at (**A**) 12,000× (scale bar = 2 µm) and (**B**) 30,000× (scale bar = 1 µm). Quantitative analyses were performed on images acquired at 12,000× and included (**C**) mitochondrial score, (**D**) mitochondrial number, (**E**) mitochondrial area (%), (**F**) mean mitochondrial area, (**G**) mitochondrial density, (**H**) mitochondrial fragmentation, and (**I**) cytoplasmic rarefaction index. Data are presented as mean ± SEM (n = 5 per group). Statistical analysis was performed using one-way ANOVA followed by Tukey’s post hoc test, except for panel E, which did not meet the normality assumption (Shapiro–Wilk test) and was analyzed using the Kruskal–Wallis test followed by Dunn’s post hoc test. * *p* ≤ 0.05 vs. CT; # *p* ≤ 0.05 vs. HF; δ *p* ≤ 0.05 vs. HF+A; + *p* ≤ 0.05 vs. HF+T. M: Mitochondria; ER: Endoplasmic reticulum.

Figure 2A,B illustrate representative images of hepatic ultrastructure. The HF (*p* < 0.0001) and HF+T (*p* = 0.0332) groups exhibited significantly higher mitochondrial damage scores (Figure 2C) compared to the CT group. All interventions reduced this parameter relative to HF (HF+A: *p* < 0.0001; HF+T: *p* < 0.0001; HF+TA: *p* < 0.0001), indicating partial preservation of mitochondrial integrity.

Mitochondrial number (Figure 2D) was significantly reduced in the HF (*p* = 0.0016) and HF+T (*p* = 0.0012) groups compared to CT. In contrast, both HF+A and HF+TA groups showed increased mitochondrial number relative to HF (*p* = 0.0480 and *p* = 0.0254, respectively) and HF+T (*p* = 0.0390 and *p* = 0.0205, respectively), suggesting enhanced mitochondrial biogenesis or turnover. No significant differences in mitochondrial area were observed among groups (Figure 2E). However, the HF+T group exhibited an increase in mean mitochondrial area (Figure 2F) compared to CT (*p* = 0.0123), which may indicate mitochondrial swelling. Consistently, mitochondrial density (Figure 2G) was reduced in HF (*p* = 0.0016) and HF+T (*p* = 0.0014) groups, whereas HF+A showed no difference from CT and a ~30% increase compared to HF, reinforcing its protective effect on mitochondrial organization.

Mitochondrial fragmentation (Figure 2H) was reduced in HF (*p* = 0.0167), HF+T (*p* = 0.0084), and HF+TA (*p* = 0.0146) groups compared to CT, suggesting impaired mitochondrial dynamics. Notably, HF+A did not differ from CT, indicating preservation of mitochondrial fission–fusion balance. However, none of the interventions significantly reversed fragmentation in HF-fed animals.

The cytoplasmic rarefaction index (Figure 2I), a marker of cellular degeneration, was significantly increased in HF (*p* < 0.0001), HF+T (*p* = 0.0002), and HF+TA (*p* = 0.0005) groups compared to CT. In contrast, HF+A showed a significantly lower rarefaction index than HF (*p* = 0.0001), HF+T (*p* = 0.0154), and HF+TA (*p* = 0.0454), with values comparable to CT, indicating marked preservation of cellular integrity.

### 2.4. TEM-Based Evaluation of ER Stress in Liver

TEM analysis (Figure 2B) qualitatively revealed marked disorganization of the endoplasmic reticulum (ER) in the HF group, characterized by dilated and fragmented cisternae, consistent with ER stress. Partial restoration of ER ultrastructure was observed in both HF+A and HF+T groups, as indicated by improved cisternal organization and reduced swelling. In contrast, the HF+TA group did not exhibit clear morphological improvement compared to the HF group, maintaining evident ER disarrangement and cisternal dilation. This finding suggests that the combined intervention was less effective in preserving ER structural integrity under the present conditions. Despite these ultrastructural alterations, no significant differences in gene expression of classical ER stress markers, including CHOP and ATF4 (Figure S2), were observed among the experimental groups. This apparent discrepancy between morphological and molecular data may reflect the operation of the Unfolded Protein Response (UPR) in its adaptive phase, in which structural remodeling of the ER can occur without necessarily engaging downstream transcriptional effectors such as CHOP and ATF4 [19]. This finding highlights the importance of integrating multiple levels of analysis when assessing cellular stress. Regarding the HF+TA group, the persistence of ER disorganization despite combined intervention may reflect a context-specific response in which increased metabolic demand imposed by training transiently sustains ER structural stress independently of transcriptional activation.

### 2.5. Mitochondrial Biogenesis and Functional Marker Analysis

**Figure 3:**
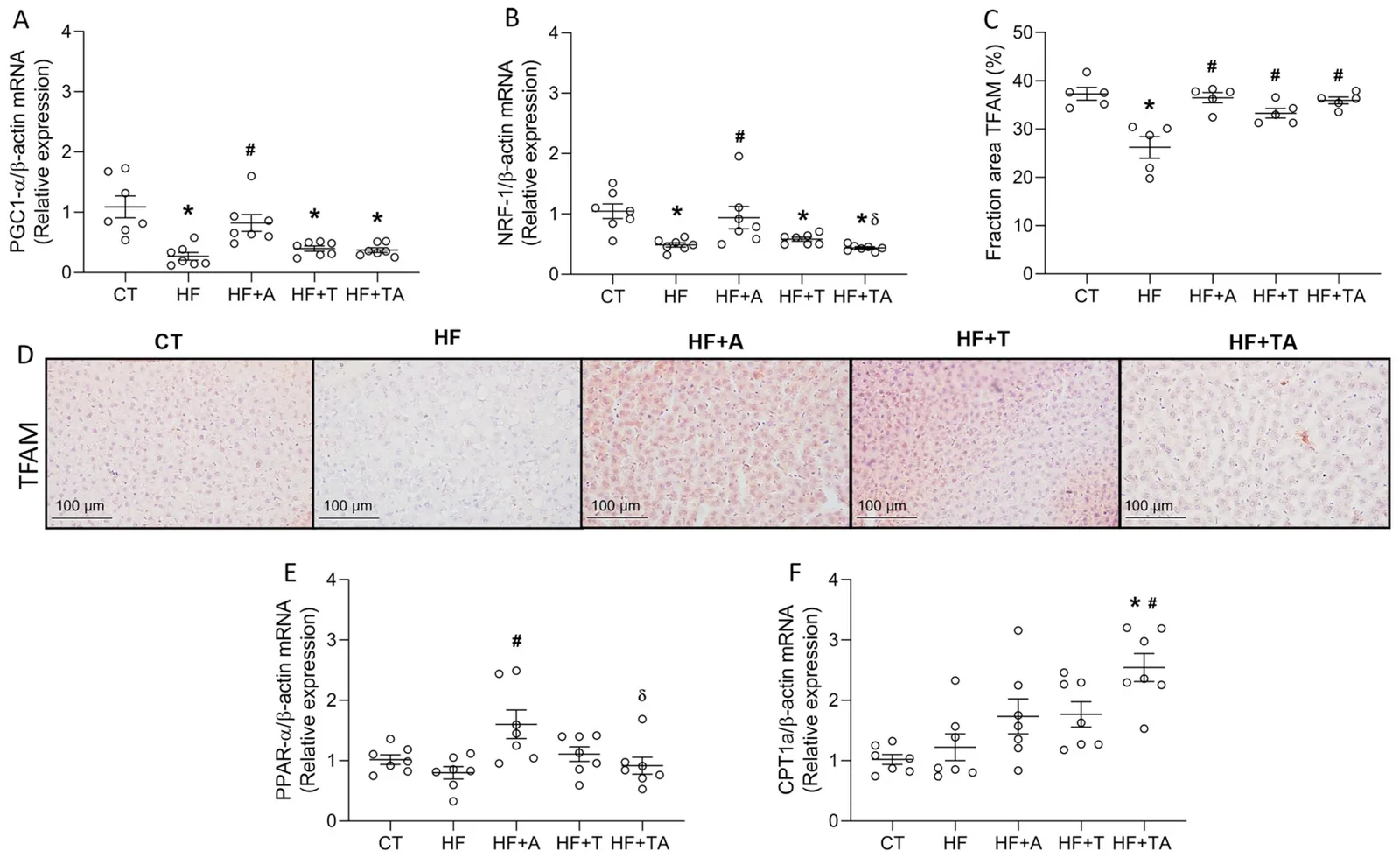
Hepatic mitochondrial biogenesis and fatty acid β-oxidation markers in high-fat-diet-fed rats following ASE and physical training. mRNA expression of the mitochondrial biogenesis markers PGC-1α (**A**) and NRF1 (**B**); TFAM protein expression assessed by immunohistochemistry with quantification of the positive staining area (**C**) and representative photomicrograph (**D**; 40×, scale bar = 50 µm); and mRNA expression of the fatty acid β-oxidation markers PPARα (**E**) and CPT1 (**F**). Data are presented as mean ± SEM (n = 7 per group for gene expression; n = 5 per group for TFAM immunohistochemistry). Statistical analysis was performed using one-way ANOVA followed by Tukey’s post hoc test. * *p* ≤ 0.05 vs. CT; # *p* ≤ 0.05 vs. HF; δ *p* ≤ 0.05 vs. HF+A.

The quantification of mRNA demonstrated a reduction in PGC1-α (Figure 3A) in the HF (*p* = 0.0001), HF+T (*p* = 0.0010), and HF+TA (*p* = 0.0006) groups compared to the CT, and only the HF+A group presented an increase in the mRNA compared to the HF group (*p* = 0.0107). Similarly, the mRNA quantification of NRF-1 (Figure 3B) showed a decrease in the HF (*p* = 0.0045), HF+T (*p* = 0.0239), and HF+TA (*p* = 0.0018) groups, and the HF+A group presented an increase in the NRF-1 mRNA compared to the HF group (*p* = 0.0292) and HF+TA group (*p* = 0.0129).

TFAM immunostaining was reduced in the HF group compared with the CT group (*p* = 0.0001), suggesting disruption of mitochondrial biogenesis. In contrast, animals from all treatment groups (HF+A: *p* = 0.0003; HF+T: *p* = 0.0118; HF+TA: *p* = 0.0005) showed increased TFAM levels compared with the HF group, indicating restoration of this pathway following the intervention. Notably, the HF+T and HF+TA groups showed increased TFAM expression, despite not showing significant changes in PGC1α and NRF1 mRNA levels, suggesting potential post-transcriptional regulation or alternative pathways contributing to the exercise-induced stimulation of mitochondrial biogenesis in hepatic tissue.

PPARα mRNA levels (Figure 3E) were quantified to evaluate β-oxidation regulation in the liver. Although no difference was observed between the CT and HF groups, the HF+A group showed an increase in PPARα compared to the HF group (*p* = 0.0046), and the association of the strategies decreased these levels compared to the HF+A group (*p* = 0.0194). Additionally, the HF+TA presented an increase in mRNA expression of CPT1α (Figure 3F) compared to the CT (*p* = 0.0002) and HF (*p* = 0.0015) groups. No differences were observed between the HF+T and HF groups.

### 2.6. Mitochondrial Respiration in Carbohydrate and Lipid Metabolism

**Figure 4:**
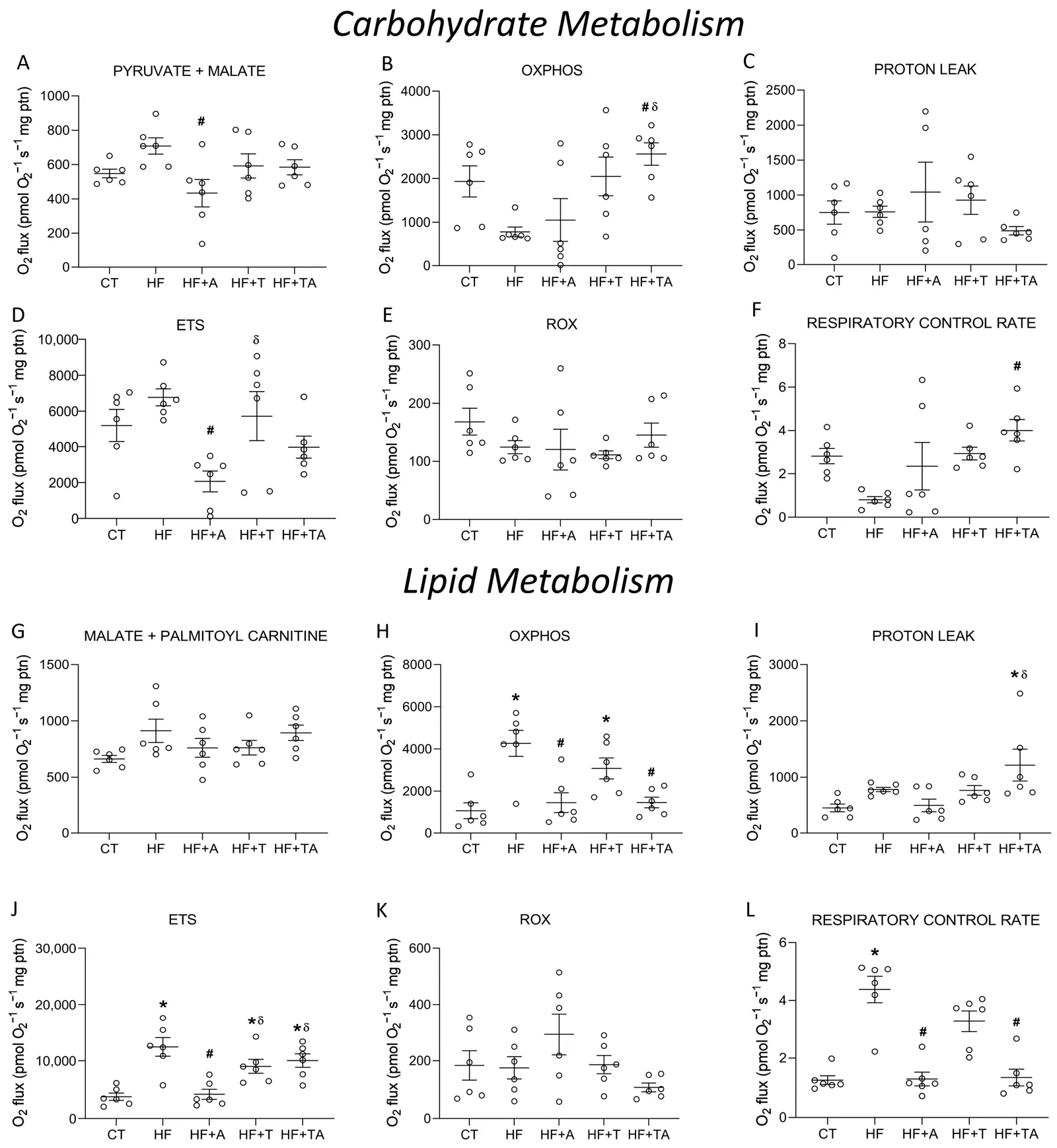
Hepatic mitochondrial respiration in high-fat-diet-fed rats following ASE and physical training. Carbohydrate-supported respiration (complex I substrates): O2 flux is shown after the addition of (**A**) pyruvate + malate, (**B**) OXPHOS, (**C**) proton leak, (**D**) ETS, (**E**) ROX, and (**F**) respiratory control ratio (RCR). Lipid-supported respiration: O2 flux is shown after the addition of (**G**) palmitoyl-carnitine + malate, followed by (**H**) OXPHOS, (**I**) proton leak, (**J**) ETS, (**K**) ROX, and (**L**) RCR. OXPHOS, ADP-stimulated respiration; ROX, residual oxygen consumption after antimycin A; ETS, maximal respiratory capacity after FCCP titration; and RCR, OXPHOS/proton leak. Data are presented as mean ± SEM (n = 6 per group, except panel (**C**), n = 5). Statistical analyses were performed using one-way ANOVA followed by Tukey’s post hoc test or, when normality was not met (panels (**B**,**E**,**F**,**L**)), the Kruskal–Wallis test followed by Dunn’s post hoc test. * *p* ≤ 0.05 vs. CT; # *p* ≤ 0.05 vs. HF; δ *p* ≤ 0.05 vs. HF+A.

In carbohydrate-supported respiration, no differences in OXPHOS were observed between HF and CT groups (Figure 4B). However, the combined intervention (HF+TA) significantly increased OXPHOS compared to both HF (*p* = 0.0317) and HF+A (*p* = 0.0480), accompanied by an increase in respiratory control ratio (RCR; *p* = 0.0093 vs. HF; Figure 4F), indicating improved mitochondrial coupling and efficiency. In contrast, the electron transport system (ETS) capacity did not differ between the HF and CT groups. However, ETS capacity was significantly reduced in the HF+A group compared with both HF (*p* = 0.0055) and HF+T (*p* = 0.0419) groups (Figure 4D), indicating a treatment-associated reduction in maximal respiratory capacity. No significant differences were observed in routine respiration (Figure 4A), proton leak (Figure 4C), or residual oxygen consumption (ROX; Figure 4E) between HF and CT groups, suggesting that basal mitochondrial function and non-mitochondrial oxygen consumption remained unchanged.

In lipid-supported respiration, the HF group exhibited increased OXPHOS and ETS capacity (*p* = 0.0004 and *p* = 0.0002, respectively) (Figure 4H,J), along with elevated RCR (*p* = 0.0117) (Figure 4L), reflecting a shift toward enhanced fatty acid oxidation. This profile likely reflects compensatory hyperactivation in response to chronic lipid oversupply, as previously described [14]. ASE treatment (HF+A) significantly reduced OXPHOS (*p* = 0.0018), ETS (*p* = 0.0003), and RCR (*p* = 0.0173) compared to HF, restoring values closer to CT levels (Figure 4H,J,L). This pattern suggests normalization of mitochondrial function rather than impairment, potentially reflecting reduced metabolic overload and improved substrate utilization efficiency. In contrast, physical training alone (HF+T) did not significantly reverse the HF-induced alterations in lipid-supported respiration. OXPHOS and ETS rates remained elevated and comparable to HF (Figure 4H,J), indicating persistence of the lipid-driven metabolic phenotype. This observation is consistent with the limited impact of training on hepatic mitochondrial biogenesis markers observed in the present study.

Interestingly, the combined intervention (HF+TA) resulted in a distinct respiratory profile. While OXPHOS was reduced compared to HF (*p* = 0.0019; Figure 4H), ETS capacity and proton leak were increased relative to CT and HF+A (Figure 4I,J). Additionally, RCR was reduced to levels comparable to those of CT (*p* = 0.0077 vs. HF, Figure 4L). This pattern may reflect adaptive mitochondrial uncoupling, potentially mediated by enhanced proton-leak pathways, which can limit ROS production by partially dissipating the mitochondrial membrane potential [20]. However, since non-specific proton leak secondary to oxidative membrane damage can produce a similar respiratory profile, and considering the persistence of mitochondrial oxidative stress markers in this group, both mechanisms may contribute to the observed effects and cannot be distinguished without direct assessment of uncoupling protein expression or membrane integrity.

### 2.7. Oxidative Status in Mitochondrial Fraction and Liver

**Figure 5:**
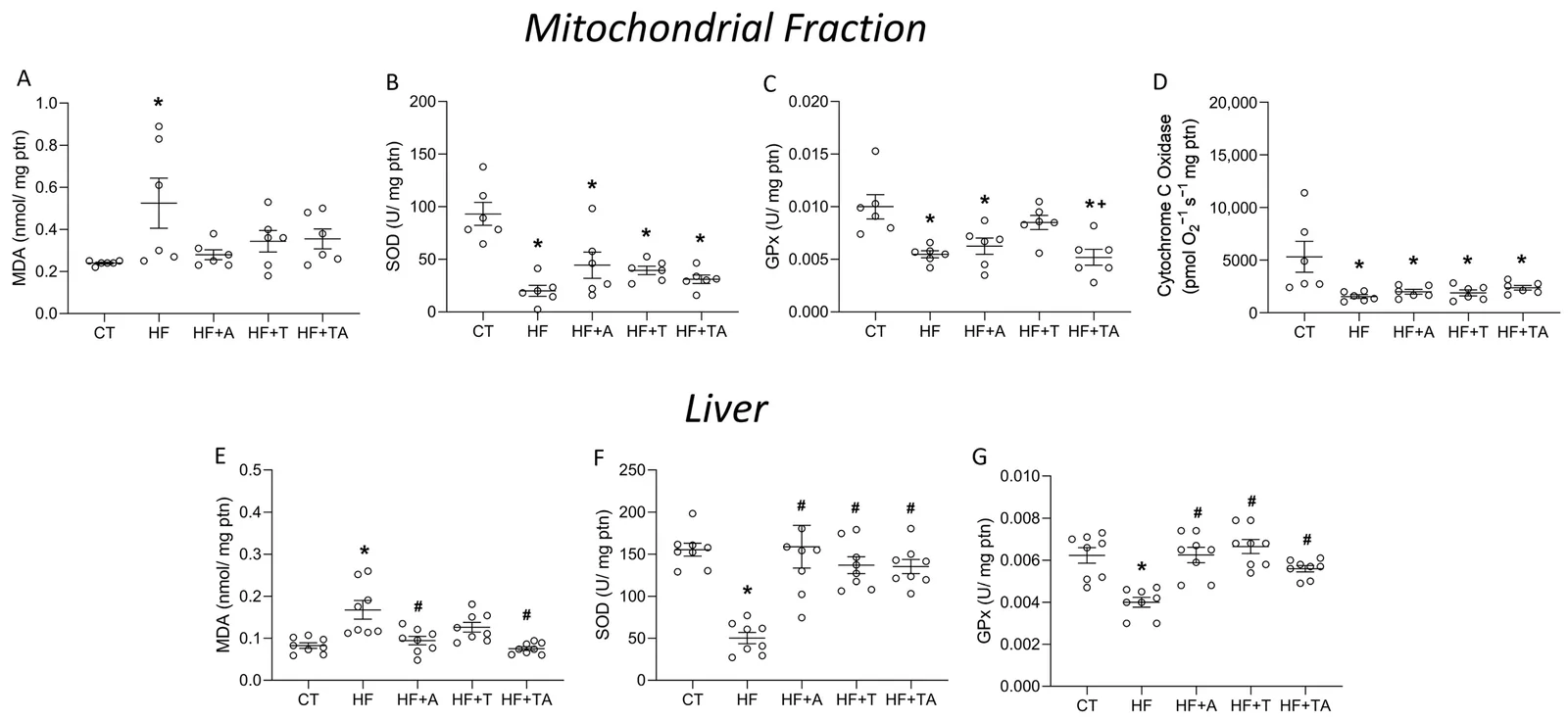
Mitochondrial and hepatic oxidative status in high-fat-diet-fed rats following ASE and physical training. In the mitochondrial fraction: (**A**) malondialdehyde content, (**B**) superoxide dismutase activity, (**C**) glutathione peroxidase activity, and (**D**) cytochrome c oxidase activity. In total liver homogenates: (**E**) malondialdehyde content, (**F**) superoxide dismutase activity, and (**G**) glutathione peroxidase activity. Data are presented as mean ± SEM (n = 6 per group for mitochondrial analyses; n = 8 per group for total liver homogenates). Statistical analysis was performed using one-way ANOVA followed by Tukey’s post hoc test. * *p* ≤ 0.05 vs. CT; # *p* ≤ 0.05 vs. HF; + *p* ≤ 0.05 vs. HF+T.

In the mitochondrial fraction, the HF group exhibited increased lipid peroxidation, as indicated by elevated MDA levels (*p* = 0.0267; Figure 5A), accompanied by a marked reduction in antioxidant enzyme activities, including SOD (*p* < 0.0001; Figure 5B) and GPx (*p* = 0.0035; Figure 5C), characterizing a state of mitochondrial oxidative stress. None of the interventions fully reversed this mitochondrial redox imbalance. MDA levels remained unchanged across treatment groups compared with the HF group (Figure 5A). SOD activity remained significantly reduced in all treated groups relative to CT (HF+A: *p* = 0.0024; HF+T: *p* = 0.0008; HF+TA: *p* = 0.0001; Figure 5B). GPx activity remained significantly lower than CT in the HF+A (*p* = 0.0184) and HF+TA (*p* = 0.0018) groups (Figure 5C). Notably, GPx activity in the HF+TA group did not differ significantly from either the CT or HF groups, suggesting a partial but incomplete recovery. These findings indicate persistent mitochondrial oxidative stress despite the applied interventions. Consistently, cytochrome c oxidase (COX) activity (Figure 5D), a key component of the electron transport chain, was reduced in all experimental groups compared to CT (HF: *p* = 0.0054; HF+A: *p* = 0.0166; HF+T: *p* = 0.0132; HF+TA: *p* = 0.0410; (Figure 5D), suggesting sustained impairment of mitochondrial respiratory capacity.

In contrast, analysis of the total liver homogenate revealed a distinct pattern. The HF group showed increased MDA levels (*p* = 0.0003; Figure 5E) and reduced antioxidant enzyme activities (SOD: *p* < 0.0001; GPx: *p* < 0.0001; Figure 5F,G), reflecting elevated global oxidative stress. Notably, ASE, either alone or combined with exercise, significantly reduced MDA levels compared to HF (*p* = 0.0019 and *p* < 0.0001, respectively), indicating attenuation of lipid peroxidation at the tissue level. Moreover, all interventions increased SOD (HF+A: *p* < 0.0001; HF+T: *p* = 0.0006; HF+TA: *p* = 0.0008) and GPx activities (HF+A: *p* < 0.0001; HF+T: *p* < 0.0001; HF+TA: *p* = 0.0055) relative to HF, suggesting restoration of hepatic antioxidant defenses.

## 3. Discussion

The use of medicinal plants and their bioactive phytochemicals has emerged as a promising strategy for managing metabolic disorders [21]. Among these, polyphenols exert protective effects by modulating mitochondrial function, biogenesis, and turnover, thereby mitigating oxidative stress and inflammation [22]. This effect is especially notable in catechins and proanthocyanidins, which are abundantly present in ASE [1,12,23]. In line with previous studies [24,25,26], both ASE and physical training attenuated body mass gain, counteracting HFD-induced metabolic disturbances and reinforcing the translational potential of açaí seed-derived polyphenols as agro-industrial by-product ingredients.

In this context, the dose used in the present study corresponds to a human equivalent dose of approximately 1.9–2.3 g/day of standardized lyophilized extract for a 60–70 kg adult [27], which is within the range evaluated in human safety studies of proanthocyanidin-rich extracts [28,29,30]. These findings therefore provide a rational basis for selecting an initial dose range in future clinical studies investigating ASE as a nutritional intervention for MASLD. Nevertheless, possible variability in extract composition, including the absence of independent quantitative HPLC characterization of individual phenolic compounds for the present batch, together with the inherent limitations of extrapolating findings from a male rodent model, highlights the need for batch standardization and well-designed clinical trials before therapeutic recommendations can be made.

HFD-induced metabolic alterations are closely linked to insulin resistance and the development of type 2 diabetes [31]. Physical training lowered glycemia both when applied alone and in combination with ASE, suggesting improved insulin sensitivity, in line with previous reports [16]. By contrast, ASE alone did not significantly alter glycemic levels, although it was more effective at attenuating hepatic steatosis, suggesting a more direct effect on liver metabolism. Notably, ASE alone normalized triglyceride levels, which were further reduced by the combined intervention, suggesting complementary effects on lipid metabolism.

The absence of cholesterol-lowering effects may reflect the distinct regulatory pathways governing cholesterol metabolism. Specifically, while PPARα activation in HF+A primarily drives mitochondrial fatty acid β-oxidation, hepatic cholesterol biosynthesis and clearance rely on independent transcriptional programs, such as SREBP-2 and LXR pathways [32]. Additionally, the six-week intervention period may have been too short to induce detectable changes in circulating cholesterol levels under sustained dietary lipid excess.

Beyond these systemic biochemical alterations, hepatic lipid accumulation was directly examined to confirm the establishment of a steatotic phenotype at the tissue level. The pronounced hepatic lipid accumulation observed in the HF group is consistent with hepatic steatosis. It highlights the metabolic impact of chronic HFD exposure and supports the establishment of a MASLD phenotype in this model [33]. ASE alone showed greater efficacy in reducing hepatic lipid deposition than exercise or their combination, consistent with its more direct effect on hepatic lipid metabolism [12]. One possible explanation is that exercise-induced fatty acid mobilization from adipose tissue may transiently enhance hepatic lipid influx [34], partially counteracting its effects on lipid oxidation. Biochemical assessment of hepatic triglyceride content further corroborated the steatotic phenotype. Moreover, it indicated that the combined intervention was the most effective approach for reducing hepatic triglyceride accumulation, suggesting complementary actions of ASE and physical training on hepatic lipid handling. Notably, only the combined intervention significantly reduced hepatic triglyceride content relative to HF, diverging from the Oil Red O pattern in which ASE alone showed greater efficacy. This difference likely reflects the distinct analytical scope of the two methods: Oil Red O staining reflects the accumulation of neutral lipid droplets, whereas the biochemical assay specifically quantifies hepatic triglyceride content. Together, these findings suggest that ASE may have a greater effect on overall hepatic neutral lipid deposition, whereas the combined intervention appears to be particularly effective in reducing hepatic triglyceride accumulation.

Collectively, the ultrastructural findings demonstrated that HFD induces profound alterations in mitochondrial structure, including reduced mitochondrial number and density, decreased fragmentation consistent with megamitochondria formation, elevated damage scores, and increased cytoplasmic rarefaction, which are hallmarks of severe mitochondrial dysfunction that disrupts electron transport chain efficiency and ATP production [17,20,35,36]. Among the interventions, ASE showed the strongest protective effect, restoring mitochondrial number and density, preserving mitochondrial fragmentation patterns, and reducing both the damage score and the cytoplasmic rarefaction index, indicating improved mitochondrial quality control and cellular homeostasis [8]. In contrast, physical training mainly reduced the damage score without fully restoring mitochondrial number, density, or dynamics, and the increased mean mitochondrial area in HF+T suggests persistent swelling and incomplete functional recovery [17,20,36]. These findings indicate that ASE exerts a more direct and robust effect on hepatic mitochondrial homeostasis than physical training, underscoring the practical value of valorizing açaí seed residues as functional ingredients for the management of metabolic disease.

Mitochondrial biogenesis is impaired in MASLD [8,37], as observed in HF-fed animals. ASE treatment was associated with upregulation of the mitochondrial biogenesis regulators PGC-1α and NRF-1, together with increased TFAM immunoreactivity, and improved mitochondrial ultrastructural integrity, findings consistent with previous reports linking polyphenol-rich extracts to PGC-1α-mediated mitochondrial biogenesis [3,38]. Although TFAM protein expression was confirmed by immunohistochemistry, the absence of Western blot quantification for PGC-1α, NRF-1, and TFAM represents a limitation of the present study. Future investigations should therefore include protein-level assessment of the full upstream regulatory pathways to complement the transcriptional and immunohistochemical findings. Increased PPARα expression in HF+A further indicates engagement of fatty acid oxidation pathways, potentially contributing to improved hepatic lipid metabolism [32]. Notably, physical training increased TFAM expression without activation of upstream regulators, suggesting post-transcriptional or systemic regulation, which may indicate that hepatic adaptations to exercise are driven predominantly by whole-body metabolic changes [38].

A key finding is that the combined intervention (HF+TA) produced a distinct molecular signature, characterized by increased CPT1α and TFAM expression, without activating PGC-1α or NRF-1. This pattern is consistent with a shift toward improved mitochondrial function and substrate utilization [39], rather than increased mitochondrial biogenesis. Although adaptive, the lack of additive effects on key upstream regulators and structural parameters suggests that the interaction between ASE and exercise is not strictly synergistic. These results suggest overlapping mechanisms, competition for metabolic pathways, or differences in temporal molecular activation.

Respirometric analysis indicated that ASE counteracts mitochondrial hyperactivation induced by HFD, thereby restoring a more physiological respiratory profile [17,20]. Importantly, the reductions in OXPHOS, ETS, and RCR in HF+A should not be interpreted as impairment but as reversal of compensatory overactivation driven by lipid excess [20,40]. In contrast, physical training alone did not modify these parameters, indicating the persistence of a lipid-driven metabolic phenotype [15]. This finding aligns with the observed limited effects of training on hepatic mitochondrial biogenesis markers. However, the combination intervention led to specific adaptations, such as increased proton leak and CPT1α expression, which may enhance fatty acid handling and reduce lipotoxic stress [39]. Support for this comes from the reduction in plasma triglycerides, observed exclusively in the combined group, indicating improved systemic lipid metabolism.

In carbohydrate metabolism, the selective increase in OXPHOS and RCR observed only in the HF+TA group suggests enhanced metabolic flexibility, enabling more efficient substrate switching [17]. This adaptation is particularly relevant in metabolic disorders, in which impaired substrate utilization represents a central feature of disease progression [1,5]. Notably, physical training alone did not significantly improve carbohydrate-supported mitochondrial respiration in the liver, despite its well-established effects in skeletal muscle [15]. These results indicate a clear tissue-specific response, suggesting that hepatic mitochondrial adaptations to exercise are predominantly indirect and may depend on systemic metabolic regulation [16,40].

The reduction in carbohydrate-linked ETS capacity in the HF+A group, observed in the absence of changes in OXPHOS, suggests that ASE did not compromise coupled oxidative phosphorylation capacity under physiological conditions. Considering the concomitant upregulation of PPARα expression in this group, this finding is compatible with a shift in mitochondrial substrate preference toward fatty acid oxidation. A well-established mechanism underlying this metabolic adaptation involves activation of the PPARα/PDK4 axis, which limits pyruvate flux through the pyruvate dehydrogenase complex and reduces the contribution of carbohydrate-derived substrates to mitochondrial electron flow, thereby promoting metabolic flexibility [32,41]. Although PDK4 expression was not evaluated in the present study, this mechanism provides a biologically plausible explanation for the observed respiratory profile. Overall, these results indicate that ASE primarily normalizes hepatic mitochondrial respiration, while their combination promotes improved substrate flexibility and fatty acid handling.

A further methodological consideration warrants discussion: using the CT group as a normative reference assumes that the respiratory phenotype of standard-diet-fed animals represents the physiological optimum across all conditions. In the context of chronic lipid excess, however, the attenuation observed in HF+A may therefore indicate reduced metabolic overload rather than suppression of respiratory capacity—a distinction requiring mechanistic investigation beyond the scope of the present study. Despite these respiratory improvements, the oxidative status analyses reveal a more complex picture: mitochondrial redox balance remains impaired across all intervention groups, indicating that structural and transcriptional improvements do not fully translate into functional redox recovery at the mitochondrial level [37,42].

Oxidative stress is closely linked to increased reactive oxygen species (ROS) production. This is particularly driven by enhanced β-oxidation and mitochondrial overload, which contribute to mitochondrial dysfunction and the progression of MASLD [22,37]. The increase in MDA, reduction in antioxidant enzymes, and decreased COX activity observed here reinforce the central role of oxidative damage in hepatic mitochondrial impairment.

Our findings reveal a dissociation between mitochondrial and extramitochondrial redox responses. Although ASE markedly improved oxidative status in the total hepatic fraction [12,24,25,43] and physical training improved antioxidant defenses at the tissue level [24,40], neither intervention fully restored mitochondrial redox balance, highlighting mitochondrial oxidative stress as a persistent and critical component of MASLD [17,22,37,42]. The failure to restore mitochondrial redox balance likely reflects polyphenols’ predominantly extramitochondrial antioxidant action [35], further constrained by the limited capacity of proanthocyanidins to cross the inner mitochondrial membrane following extensive intestinal metabolism [44].

Similarly, physical training improved antioxidant defenses at the tissue level [24,40] but did not attenuate mitochondrial oxidative stress, suggesting that hepatic redox adaptations to exercise are largely mediated by cytosolic or anti-inflammatory pathways [45], consistent with the incomplete recovery of COX activity across all groups. The persistence of megamitochondria and cytoplasmic rarefaction in the HF group, together with the incomplete structural recovery in exercise-treated animals, further supports the notion that direct biochemical modulation by polyphenols may be more effective than systemic physiological adaptations in preserving hepatic mitochondrial integrity [17].

This study has some limitations that should be considered when interpreting the present findings. First, mitochondrial respiration was assessed using a mitochondrial-enriched fraction obtained by differential low-speed centrifugation rather than permeabilized liver fibers or highly purified mitochondria. Although the respiratory control ratio values and the consistency among the respirometric, ultrastructural, and molecular findings support the biological validity of the measurements, future studies should compare this approach with higher-resolution isolation methods. Second, the absence of intervention groups receiving a standard diet limits the ability to distinguish between the reversal of HFD-induced alterations and the direct physiological effects of ASE or aerobic training. Third, although ASE improved hepatic antioxidant status, mitochondrial oxidative stress remained elevated, suggesting that restoration of mitochondrial redox homeostasis may require longer interventions or alternative dosing and exercise protocols. Finally, ASE and aerobic training only partially preserved endoplasmic reticulum morphology, highlighting the need for further investigation of their effects on endoplasmic reticulum homeostasis, including unfolded protein response signaling and protein-folding capacity. Future studies that use dose–response and extraction optimization will help clarify the mechanisms involved. They may also support practical valorization of açaí seed by-products into standardized, scalable functional ingredients.

## 4. Materials and Methods

### 4.1. Preparation of Açaí (Euterpe oleracea Mart.) Seed Extract and Polyphenol Analysis

Açaí fruits (*Euterpe oleracea* Mart.) were obtained from the Amazon Bay region (Pará State, Brazil). Botanical identification was performed by the Goeldi Museum (Belém, Brazil), where a voucher specimen (MG 205222) was deposited.

Açaí seed extract (ASE) was prepared as previously described [11]. Briefly, açaí seeds were manually ground, boiled in distilled water (5 min), cooled to room temperature, and extracted by maceration in a 1:1 (*v*/*v*) water–ethanol mixture. The mixture was kept in amber flasks at 4 °C, with daily agitation, for 10 days. The extract was filtered under reduced pressure, concentrated by evaporation at 50–60 °C to remove ethanol, lyophilized, and stored at −20 °C until use. The extraction yield was approximately 5 g of lyophilized extract per 100 g of seeds. Batch consistency was verified by total polyphenol content using the Folin–Ciocalteu method [46], resulting in 200 mg gallic acid equivalents (GAE)/g of extract, and by confirmation of vasodilatory biological activity in rat mesenteric arterial beds (ID_50_ = 2.56 mg/mL), as previously described [11]. The analysis of the aqueous fraction residue from ASE by high-performance liquid chromatography (HPLC) and MALDI-TOF mass spectrometry was previously reported by our group [12]. The HPLC analysis of ASE revealed that it is composed of proanthocyanidins (88% of the total area) and, to a minor extent, catechin and epicatechin. The chemical and spectrometric analysis revealed that ASE is composed predominantly of polymeric procyanidins, heteropolymers with one gallocatechin unit, and, to a minor extent, of galloylated procyanidins (Figure S3).

### 4.2. Experimental Design

The study protocol was approved by the Ethics Committee for Animal Use (CEUA) at Rio de Janeiro State University (protocol no. 009/2022, 14 April 2022). Male Sprague–Dawley rats (*Rattus norvegicus*) were provided by the animal facility of the Department of Pharmacology (IBRAG/UERJ). Animals were housed in polypropylene cages (4 per cage) with untreated wood shavings as bedding under controlled conditions (21 ± 2 °C; 12 h light–dark cycle, lights on at 6:00 a.m.), with free access to food and water. After an acclimatization period, four-week-old rats were maintained on their respective diets for 10 weeks. At the start of the intervention period, animals weighed 320–450 g and were randomly allocated into five experimental groups (n = 8 per group): Control (CT), high-fat (HF), High-fat + ASE (HF+A), high-fat + training (HF+T), and high-fat + training + ASE (HF+TA). The CT group received a standard diet (10% of the energy from lipids), whereas the HF groups were fed an HFD (55% of the energy from lipids) for 16 weeks. Diets were produced by PragSoluções (São Paulo, Brazil) following AIN-93M recommendations (Table S1).

Following the initial 10 weeks of dietary intervention, ASE treatment and physical training were initiated and maintained for 6 weeks, as previously described [14]. Physical training was performed on a motorized rodent treadmill (AVS Projetos^®^, São Paulo, Brazil) for 30 min/day, five days/week, at an intensity individually prescribed as 60–70% of each animal’s maximum speed determined by a progressive maximum speed test (MST; detailed protocol in Supplementary Materials). Inter-individual variability in maximum treadmill speed at MST-1 was low across trained groups (coefficient of variation = 13.36%), and training intensity was individually prescribed as 60–70% of each animal’s own maximum speed, providing a physiologically equivalent relative exercise stimulus across animals. ASE was administered by oral gavage (200 mg/kg/day) to the HF+A and HF+TA groups, while CT, HF, and HF+T animals received an equivalent volume of water. The ASE dose was based on previous studies from our group, which used a HF diet model in rat [24]. These studies demonstrated metabolic alterations and significant improvements in parameters associated with metabolic syndrome following chronic administration of the extract. The dose of ASE administered, based on body surface area normalization [27], corresponds to a human equivalent dose (HED) of approximately 32.4 mg/kg/day, representing an estimated daily intake of 1.9–2.3 g/day for a 60–70 kg adult of a standardized lyophilized extract. To avoid acute exercise effects, all experimental procedures, including euthanasia and tissue collection, were performed 48 h after the last training session. Importantly, male rats were used exclusively to minimize variability due to hormonal cycling, and thus, caution is warranted when extrapolating the findings to females.

### 4.3. Body Weight Measurement, Feed Efficiency and Biochemistry Parameters

Body mass gain was determined by subtracting the body weight recorded after 10 weeks of high-fat-diet (HFD) feeding (baseline) from the final body weight measured at the end of the experimental period. Feed efficiency was calculated as body mass gain (g) divided by cumulative energy intake (kcal), using the respective caloric densities of the experimental diets (3.8 kcal/g for the standard diet and 5.2 kcal/g for the HFD) [47]. Following an overnight fast of 12 h, blood glucose concentration was measured from tail vein blood samples using a portable glucometer (Accu-Chek Active^®^, Roche, Basel, Switzerland). Plasma concentrations of triglycerides and total cholesterol were quantified by enzymatic colorimetric assays using commercially available kits (Bioclin, Belo Horizonte, Brazil; catalog numbers K-117 and K-083). To assess hepatic triglyceride content, liver tissue (50 mg) was homogenized in isopropyl alcohol (1 mL), sonicated for 8 min (40% amplitude, 10 s on/15 s off pulses), and centrifuged (2000× *g*, 10 min, 4 °C). The supernatant was subjected to enzymatic colorimetric quantification using the same kit described above (Bioclin, catalog number K-117) [12].

### 4.4. Euthanasia and Tissue Extraction

At the end of the treatment period (16 weeks), animals were anesthetized with isoflurane (4% for hypnosis with 1.0 L/min O_2_, followed by 2% for maintenance with 0.8 L/min O_2_). Blood was collected by abdominal aortic puncture, followed by liver excision. Following collection, blood samples were centrifuged (2000× *g*, 10 min, 4 °C), and the resulting plasma was collected for subsequent analyses.

### 4.5. High-Resolution Respirometry and Cytochrome c Oxidase Activity

Mitochondrial respiration was assessed using high-resolution respirometry (Oroboros Oxygraph-2k, Innsbruck, Austria). Fresh liver tissue (~1 g) was disrupted in MiR05 mitochondrial respiration medium using an Ultra-Turrax homogenizer (IKA^®^ T25 digital, Staufen, Germany) and subjected to three sequential centrifugation steps (1000× *g*, 10 min, 4 °C) to obtain a mitochondrial-enriched fraction. The supernatant was reserved for biochemical assays of oxidative stress. The final pellet was resuspended in MiR05 buffer, and 100 µL was added to the Oxygraph-2k chamber containing 2 mL of MiR05 buffer at 37 °C for respirometric assessment. The experimental protocol was adapted from [48] and involved sequential addition of respiratory substrates and inhibitors to evaluate mitochondrial function. Electron flux through complex IV (cytochrome c oxidase, COX) was measured in intact mitochondria after inhibition of complex III with antimycin A, and subsequent addition of tetramethyl-p-phenylenediamine (TMPD) and ascorbate. Oxygen consumption attributed to TMPD/ascorbate auto-oxidation was corrected by subtracting the residual oxygen flux measured in the presence of potassium cyanide (KCN).

### 4.6. Histological and Immunohistochemical Analysis of Frozen Liver Sections

Frozen liver samples were embedded in Tissue-Tek^®^ O.C.T.™ Compound (Sakura Finetek, Torrance, CA, USA) and cryosectioned at 10 µm using a cryostat (Leica Microsystems, Wetzlar, Germany). Sections were then collected onto glass slides. Before staining, slides were allowed to reach room temperature and subsequently fixed in 4% paraformaldehyde to preserve tissue architecture and antigen integrity. Hepatic lipid content was evaluated by Oil Red O’ staining as previously described [49]. Briefly, the slides were treated in propylene glycol (Synth, Diadema, SP, Brazil) and followed by incubation in a 60 °C preheated Oil Red O’ solution (Sigma-Aldrich, St. Louis, MO, USA). The slides were differentiated using 70% isopropanol, followed by hematoxylin counterstaining of the nuclei. Subsequently, the sections were immersed in 85% propylene glycol and mounted with a glycerin-based aqueous mounting medium (Proquímios, Rio de Janeiro, RJ, Brazil).

For immunohistochemical analysis, sections were first incubated with 0.3% hydrogen peroxide (H_2_O_2_; Proquímios, Rio de Janeiro, RJ, Brazil) to quench endogenous peroxidase activity. Next, tissue permeability was increased using 0.3% PBS containing Triton X-100, followed by blocking of non-specific binding sites with 5% bovine serum albumin (BSA; Sigma-Aldrich, St. Louis, MO, USA). To detect TFAM, the sections were incubated with the primary antibody (1:100; Boster Biological Technology, Pleasanton, CA, USA; Cat. No. PB9447) overnight at 4 °C under humidified conditions. Signal detection was achieved using a biotin–streptavidin amplification system (Vectastain Universal Quick Kit; Vector Laboratories, Newark, CA, USA), followed by visualization with 3,3′-diaminobenzidine (DAB; Dako Cytomation, Glostrup, Denmark), and the sections were counterstained with hematoxylin.

Representative images were obtained with an Olympus BX60 bright-field microscope (Olympus, Tokyo, Japan) fitted with an Olympus DP71 digital camera using a 40× objective. Digital images were captured through CellSens software (v4.2; Olympus/Evident, Tokyo, Japan). Image processing and quantification were performed in ImageJ (v1.53; NIH, Bethesda, MD, USA) using the Color Deconvolution plugin. Oil Red O staining was evaluated by measuring the red color component, whereas immunohistochemical staining was quantified from the intensity of the DAB reaction product.

### 4.7. Transmission Electron Microscopy (TEM)

Liver fragments were prepared, stained, and imaged as previously described [50]. Micrographs (12,000×) were analyzed using ImageJ (v1.53, NIH, USA). The total image area was defined with the “Rectangular selection” tool, and mitochondria were manually identified based on ultrastructural features. Their number and individual areas were measured using the “Polygon selection” tool. Total mitochondrial area per field was obtained by summing individual areas; the mean mitochondrial area was then calculated by dividing the total area by the mitochondrial count. Measures of mean mitochondrial area, density, and fragmentation (number/total area) were adapted from previous studies [51,52]. Mitochondrial morphology was scored from 0 to 4 based on structural integrity [53]: 0—intact mitochondria with visible double membrane and organized cristae; 1—early cristae disorganization with mild swelling; 2—evident swelling with partial cristae loss; 3—severe swelling and dispersed or absent cristae; 4—severely damaged mitochondria or membrane rupture. Cytoplasmic rarefaction was assessed following established ultrastructural criteria for transmission electron microscopy, with scores defined by cytoplasmic density, organelle preservation, and vacuolization: 0—dense cytoplasm, well-preserved organelles; 1—mild cytoplasmic rarefaction; 2—presence of small vacuoles or partial organelle loss; 3—extensive vacuolization, intense rarefaction, and structural disruption. All analyses were performed blinded, using multiple random fields per sample.

### 4.8. Real Time PCR Analysis

Total RNA was isolated from approximately 60 mg of liver tissue using the PureLink RNA Mini Kit (Invitrogen, Waltham, MA, USA), according to the manufacturer’s instructions. RNA yield and purity were determined by spectrophotometric analysis using a NanoVue Plus spectrophotometer (GE Healthcare, Chicago, IL, USA). First-strand complementary DNA (cDNA) was generated from 100 ng of total RNA using the High-Capacity RNA-to-cDNA Kit (Applied Biosystems, Foster City, CA, USA) in a Veriti Thermal Cycler (Applied Biosystems).

Quantitative real-time PCR (qPCR) was carried out using a Rotor-Gene Q system (Qiagen, Hilden, Germany) with SYBR Green detection chemistry (Promega, Madison, WI, USA), and fluorescence signals were monitored in the green channel. Relative gene expression levels were calculated using the 2^−ΔΔCt^ method, with β-actin serving as the endogenous control. Primer sequences are provided in Table S2.

### 4.9. Oxidative Stress Determination

Oxidative damage and antioxidant status were assessed in both whole liver homogenates and mitochondrial fractions using spectrophotometric methods. To evaluate lipid membrane damage, levels of lipid peroxidation products, specifically malondialdehyde (MDA) [54], were quantified. In addition, antioxidant defenses were assessed by measuring the activities of superoxide dismutase (SOD) [55] and glutathione peroxidase (GPx) [56]. All measurements were obtained using an Amersham Ultrospec 2100 pro spectrophotometer (GE Healthcare, Chicago, IL, USA) and normalized to each sample’s total protein content to correct for differences in protein concentration.

### 4.10. Statistical Analysis

The data are expressed as the means ± standard error of the means (SEM). Normality was assessed using the Shapiro–Wilk test. For normally distributed data, a one-way ANOVA followed by Tukey’s post hoc test was applied. Non-parametric data were analyzed using the Kruskal–Wallis test followed by Dunn’s test. GraphPad Prism version 8.0 was utilized to perform the analyses and create the graphics (GraphPad Software, San Diego, CA, USA). Statistical significance is indicated by *p* values, with *p* < 0.05 considered significant.

## 5. Conclusions

This study provides evidence that improved hepatic mitochondrial function represents an important mechanism underlying the hepatoprotective effects of açaí seed extract (ASE) in an experimental model of MASLD. ASE preserved mitochondrial integrity, upregulated key regulators of mitochondrial biogenesis, and restored mitochondrial bioenergetics. These findings support further investigation of polyphenol-rich açaí seed extract as a candidate nutritional intervention targeting mitochondrial dysfunction, a key driver of MASLD progression. The distinct effects observed with aerobic training further suggest that nutritional and lifestyle interventions may act through complementary pathways, highlighting the importance of integrated strategies for metabolic disease management. These findings provide a mechanistic rationale for future clinical studies investigating açaí seed-derived polyphenols as candidate nutritional interventions targeting mitochondrial dysfunction in MASLD. Beyond their biological relevance, they also highlight the potential of açaí seed valorization as a sustainable source of high-value bioactive ingredients, supporting nutraceutical development and circular bioeconomy initiatives.

## Figures and Tables

**Table 1:** Effects of ASE and physical training on biometric and biochemical parameters.

	CT	HF	HF+A	HF+T	HF+TA
Body mass gain (g)	25.63 ± 4.67	50.00 ± 2.67 *	19.38 ± 3.20 ^#^	31.25 ± 1.83 ^#^	14.38 ± 2.75 ^#+^
Feed Efficiency (%)	4.35 ± 0.81	11.09 ± 1.45 *	4.12 ± 0.70 ^#^	6.18 ± 0.43 ^#^	2.83 ± 0.47 ^#^
Final blood glucose (mg/dL)	80.50 ± 1.65	95.13 ± 3.10 *	93.00 ± 0.73 *	83.50 ± 2.38 ^#δ^	87.38 ± 2.20
*Plasma*					
Total cholesterol (mg/dL)	53.42 ± 2.88	77.36 ± 1.89 *	71.86 ± 2.93 *	69.50 ± 2.33 *	70.41 ± 3.63 *
Triglycerides (mg/dL)	41.11 ± 3.71	74.21 ± 8.53 *	54.44 ± 3.36	71.73 ± 6.30 *	44.09 ± 7.86 ^#+^

Values are presented as means ± SEM (n = 8 for all groups). Data were analyzed using one-way ANOVA followed by Tukey’s post hoc test and considered significant when *p* ≤ 0.05. * Significantly different from the control group; # significantly different from the HF group; + significantly different from the HF+T group; ^δ^ significantly different from the HF+A group.

## Data Availability

The original data presented in the study are openly available in FigShare at https://doi.org/10.6084/m9.figshare.32099056.

## Supplementary Materials

The following supporting information can be downloaded at: https://www.mdpi.com/article/10.3390/molecules31162925/s1. The supplementary material includes additional methodology and complementary results related to the Maximal Stress Test (MST) and ER stress analysis. Figure S1: Physical Performance; Figure S2: Analysis of ERS markers; Figure S3: HPLC analysis and MALDI-TOF mass spectrum of ASE [57,58]; Table S1: Diets composition; Table S2: List of primers used for quantitative real-time PCR analysis.

## Abbreviations

ASE	Açaí Seed Extract
ATF4	Activating Transcription Factor 4
ATP	Adenosine Triphosphate
cDNA	Complementary DNA
Cox	Cytochrome c Oxidase
CHOP	C/EBP homologous protein
CPT1a	Carnitine Palmitoyltransferase 1a
ER	Endoplasmic Reticulum
ETS	Electron Transport System
FA	Fatty Acid
GPx	Glutathione Peroxidase
HRR	High Resolution Respirometry
KCN	Potassium Cyanide
MASLD	Metabolic Dysfunction-Associated Steatotic Liver Disease
MDA	Malondialdehyde
MST	Maximal Stress Test
NRF-1	Nuclear Respiratory Factor 1
OXPHOS	Oxidative Phosphorylation
PGC1-α	Peroxisome Proliferator-Activated Receptor γ Coactivator 1-α
PPARα	Peroxisome Proliferator-Activated Receptor α
RCR	Respiratory Control Rate
RNA	Ribonucleic Acid
ROS	Reactive Oxygen Species
ROX	Residual Oxygen Consumption
SOD	Superoxide Dismutase
TEM	Transmission Electron Microscopy
TMPD	N,N,N’,N’-Tetramethyl-p-phenylenediamine
