# Supplementary material for: Polyphenol-Rich Euterpe oleracea Mart. Seed Extract Improves High-Fat-Diet-Induced MASLD Through Modulation of Hepatic Mitochondrial Respiration, Biogenesis, and Redox Homeostasis
Source: Molecules. 2026 Aug 21;31(16):2925. doi: 10.3390/molecules31162925 (PMC13515159; doi:10.3390/molecules31162925)
Supplement: Supplementary file 1 [file molecules-31-02925-s001.zip › molecules-4452350-supplementary.pdf]

Supplementary Material

1. Supplementary Results

Performance in the MST

The physical performance was analyzed based on the time (Figure S1A) and distance (Figure S1B) covered by the animals during the MST sessions. In the first MST, there was no significant difference between the groups in time and distance. In the second and third tests, animals in the HF+T and HF+TA groups showed improved performance compared to their respective baseline tests. From the second to the first test, the HF+T group showed significant increases in both time and distance ($p = 0.0050$ and $p = 0.0046$, respectively), and similar results were observed in the HF+TA group (time: $p = 0.0042$; distance: $p = 0.0048$). This pattern was maintained in the third test, with both HF+T and HF+TA groups exhibiting higher time ($p<0.0001$) and distance ($p < 0.0001$) values compared to their first test. In contrast, a decline in performance was observed in the HF group during the final test, with reductions in both distance and time (time: $p = 0.0253$; distance: $p = 0.0261$) compared to the first test.

Differences between the experimental groups were observed in the HF+T and HF+TA groups compared to the HF group in the second (HF+T: $p = 0.0038$; HF+TA: $p = 0.0041$) and third tests ($p \leq 0.0001$ for both comparisons), with positive interactions in the group and time ($p < 0.0001$, Two-Way ANOVA). However, no significant difference was found between HF+T and HF+TA.

Transcriptional Profile of ERS Markers

As a complement to the ultrastructural analysis of the ER by MET, the mRNA expression of key ERS markers was also evaluated. However, no statistically significant differences were observed in CHOP (Supplementary Figure 2A) or ATF4 (Supplementary Figure 2B) expression among the experimental groups, including the HF+TA group, which showed distinct ultrastructural features.

Figure S1. Physical Performance.

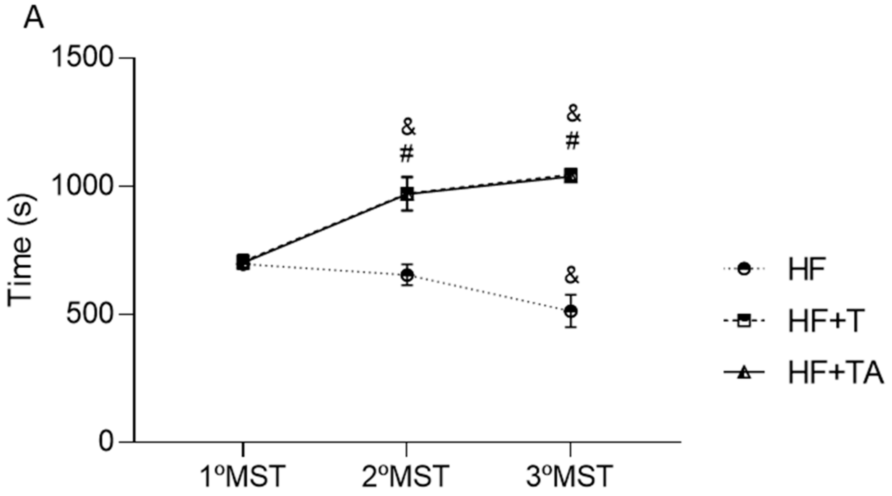

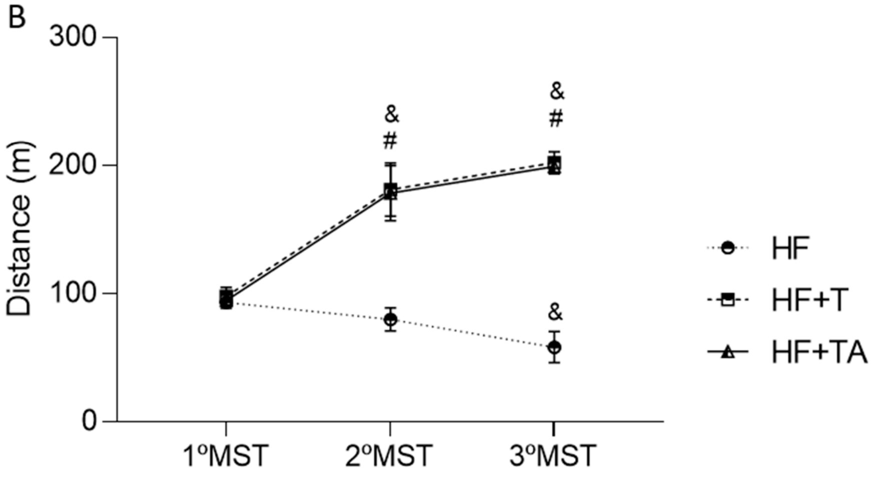

Legend: Parameter of time (A) and distance (B) during the Maximal Stress Test (MST) in HF-fed rats. Differences between groups were analyzed using two-way ANOVA followed by Tukey's post hoc test (#Significantly different from the HF group), and differences from the 1st MST were analyzed by paired Student's t-test (& significantly different from the 1st MST). Values are expressed as mean ± SEM (n = 8) and considered significant when $p \leq 0.05$.

Figure S2. Analysis of ERS markers.

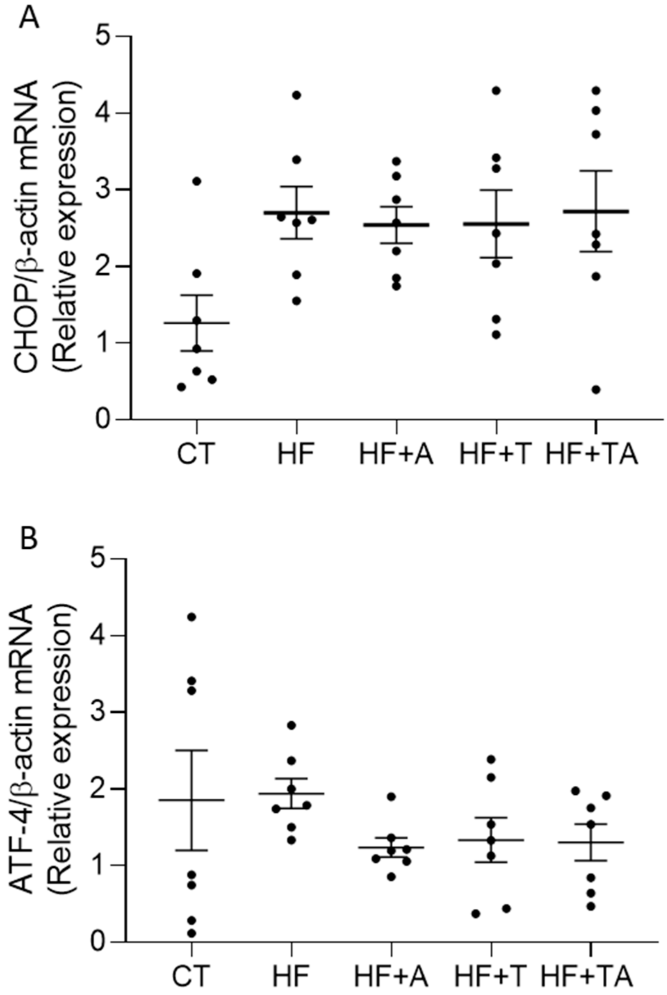

Legend: Effects of ASE and physical training on mRNA expression of Endoplasmic Reticulum Stress genes CHOP (A) and ATF4 (B) in liver from HF-fed rats. Data were analyzed using one-way ANOVA followed by Tukey's post hoc test. Values are presented as means ± SEM (n = 7) and considered significant when $p \leq 0.05$.

2. Supplementary Methods

Institutional Review Board Statement

All experimental procedures were approved by the Ethics Committee for Experimental Animals Use and Care (CEUA) of the Institute of Biology/Rio de Janeiro State University (protocol: CEUA nº 009/2022, date: 14/04/2022) and were carried out in compliance with the guidelines of the National Institutes of Health guide for the care and use of Laboratory animals (NIH Publications No. 8023, revised 1978), and the National Council for Animal Experimentation Control (CONCEA). All applicable international, national, and/or institutional guidelines for the care and use of animals were followed.

The present study was designed to evaluate the effects of ASE and physical training specifically in the context of HFD-induced metabolic dysfunction. The inclusion of treated groups under standard dietary conditions was beyond the scope of the present experimental design and represents a limitation to be addressed in future studies. Sample size determination was informed by previous studies from our research group employing similar experimental designs.

Figure S3. HPLC analysis and MALDI-TOF mass spectrum of ASE

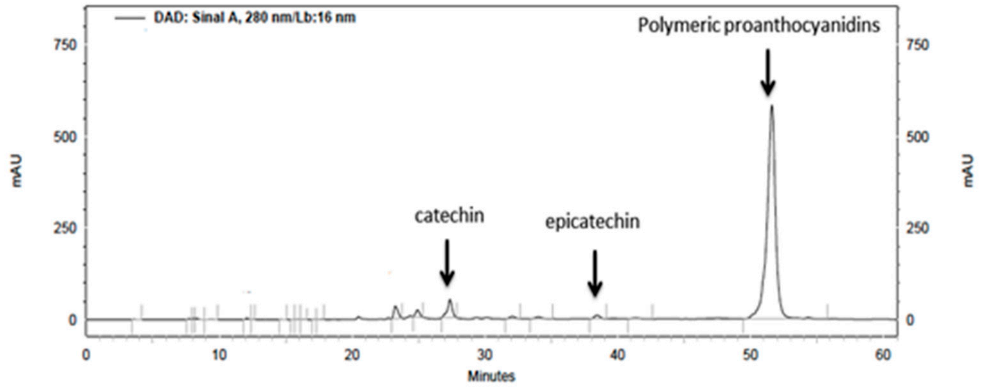

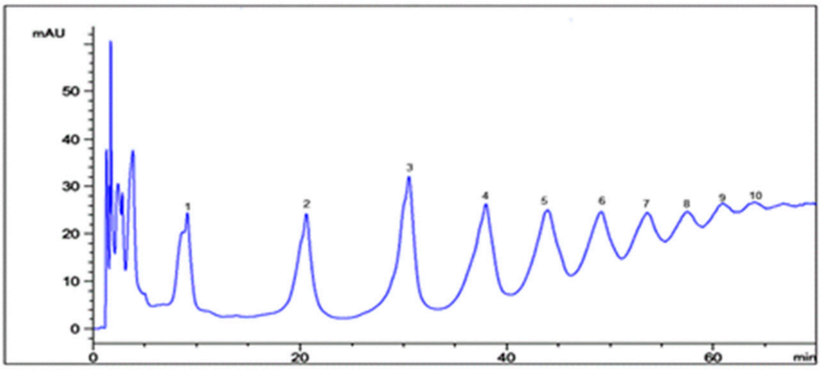

HPLC analysis of ASE. (A) HPLC analysis of the aqueous residue of ASE according to Peng, et al (2001) [57]. The marked peaks of 27, 38 and 51 min correspond to catechin, epicatechin and oligomeric and polymeric condensed tannins. (B) HPLC analysis of the aqueous fraction residue according to Kelm, et al (2006) [58], where the marked peaks (1 to 10) shows the series of condensed tannins present in the sample from monomer to decamer.

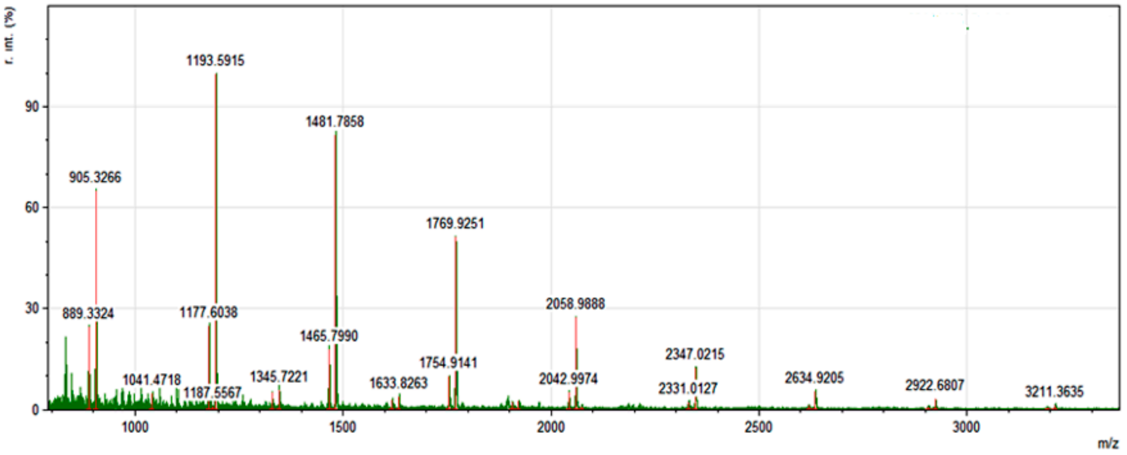

MALDI-TOF mass spectrum of the aqueous fraction residue from ASE. The spectrum was obtained on a Bruker Autoflex Speed; DHB matrix, 1:1:10 (sample: NaCl:matrix). Mass peaks are of sodium adducts of B-type proanthocyanidins: [M + Na] + m/z = 889, 903, 1177, 1193, 1465, 1481, 1753, 1769, 2041, 2058, 2329, 2347, 2617, 2634, 2907, 2922, 3193 and 3211.

References

[57] Peng Z, Hayasaka Y, Iland PG, Sefton M, Høj P, Waters EJ. (2001) Quantitative analysis of polymeric procyanidins (Tannins) from grape (Vitis vinifera) seeds by reverse phase high-performance liquid chromatography. *Journal of Agricuntural Food Chemistry,* **49(1)**: 26–31.

[58] Kelm MA, Johnson JC, Robbins RJ, Hammerstone JF, Schmitz HH. (2006) High-performance liquid chromatography separation and purification of cacao (Theobroma cacao L.) procyanidins according to degree of polymerization using a diol stationary phase. *Journal of Agricuntural Food Chemistry*, **54**: 1571–1576.

Maximum Stress Test (MST) and Treadmill Exercise Training

At the end of the 10th week of HFD exposure, the animals from the HF group and HF+T groups were submitted to the first maximal stress test (MST) using a rodent motorized treadmill with eight independent lanes (AVS Projetos®, São Paulo, Brazil). Each rat was tested individually on the treadmill, with its tail held throughout the test to maintain gait during the run. The MST started at 3 m/min and increased by 4 m/min every three minutes until the animal reached exhaustion. Exhaustion was defined by specific signs, including loss of coordinated gait, tail stiffening, and retraction of the ears, at which point the test was immediately stopped. This test determined the training speed of the animals during the experimental protocol, which was set at 60% to 70% of the maximal velocity obtained during an MST at 0% grade. The chronic physical training was performed over the next six weeks, consisting of 30 minutes per day, five days a week. The rats underwent a warm-up period, consisting of running and recovery, during the six weeks of the experimental protocol, as previously described (Soares et al., 2020). Two more MST were performed during the protocol: at the end of the third week of treatment to adapt the experimental protocol and at the end of the sixth week to evaluate the physical performance.

Sample sizes and exclusions

Sample size for the overall experimental design was n = 8 animals per group. For histological analyses, a subset of n = 5 animals per group was used, in accordance with the standard sample size routinely adopted by our research group for this type of analysis, given the homogeneity typically observed in histological outcomes. For gene expression analysis by RT-qPCR, n = 7 animals per group were used, corresponding to the maximum number of samples that could be run in duplicate within a single 72-well plate, thereby avoiding inter-run variability associated with splitting samples across plates. For analyses performed in freshly isolated mitochondria, n = 6 animals per group were used, reflecting the maximum number of samples that could be processed simultaneously during euthanasia, given logistical constraints inherent to working with fresh tissue. No animals or data points were excluded from any analysis. For the high-resolution respirometry protocol performed in isolated mitochondria, all parameters were obtained from a single sequential substrate-uncoupler-inhibitor titration (SUIT) run per sample. For the

parameter of proton leak under carbohydrate-supported respiration, data from one animal were not available (n = 5) due to a technical issue confined to that specific step of the titration protocol; data from the same animal were successfully obtained for all other respiratory parameters within the same run, indicating that the issue was specific to this measurement point rather than reflecting a problem with overall sample or run quality.

Statistical Analysis

The data are expressed as the means ± standard error (SEM) of the means. All data were first tested for normality using the Shapiro–Wilk test. Variables influenced by a single factor (e.g., PCR results) were analyzed using one-way ANOVA followed by Tukey's post hoc test. For outcomes influenced by two factors, such as time and group in the Maximum Stress Test, a two-way ANOVA was applied, also followed by Tukey's post hoc test when appropriate. Additionally, to evaluate the effect of time within the same group (e.g., first vs. final test in the HF group), a paired Student's t-test was used. Statistical significance was set at P values < 0.05.

Table S1 – Diets composition.

Nutrients (U/Kg diet)	Standart Diet	High-fat Diet
Casein (g)	140	180
Corn strach (g)	465,7	147,6
Starch dextrinized (g)	155	155
Soybean oil (g)	40	40
Lard (g)	-	278
Microcrystal cellulose (g)	50	50
L-cystine (g)	1,8	1,8
Choline chloride	2,5	2,5
BHT (g)	0,008	0,008
Mineral mix (g)	35	35
Vitamin mix (g)	10	10
Energy (kcal)	3.800	5.200
Carbohydrate (%)	**75**	**31**
Protein (%)	**14**	**14**
Lipids (%)	**10**	**55**

Composition of the standard and high-fat diets offered to the animals during the experimental protocol. Balanced diet by Prag Solutions. Vitamins and minerals follow the AIN-93M standard recommended for rodents.

Table S2 – List of primers used for quantitative real-time PCR analysis.

Gene	*Forward (5' to 3')*	*Reverse (5' to 3')*
**ATF4**	GACCGAGATGAGCTTCCTGAACAG	CCGCCTTGTCGCTGGAGAAC
**CHOP**	GGAGAAGGAGCAGGAGAATG	GAGACAGACAGGAGGTGATG
**CPT1a**	AAGCTGTGGCCTTCCAGTTC	GGATGAAATCACACCCACCA
**NRF1**	TGTTTGAGTCTAACCCATCTATCCG	TGCTGTCCCACTCGTGTCGT
**PGC-1α**	GACCACAAACGATGACCCTC	TGTTGCGACTGCGGTTGT
**PPARα**	CGTTCGCAGCTGTTTTGTGG	TGGCTCGCCTCTAAGATCCC
**β-actin**	CGCGAGTACAACCTTCTTGCAG	CGCAGCGATATCGTCATCCA

Sequences of real-time PCR primers. ATF4: activating transcription factor 4; CHOP: C/EBP homologous protein; CPT1a: carnitinepalmitoyl transferase 1a; NRF1: Nuclear respiratory factor 1; PGC-1α: peroxisome proliferator-activated receptor gamma coactivator 1-alpha; PPARα: peroxisome proliferator-activated receptor-alpha; β-actin: endogenous housekeeping gene.
